# Prevalence of ACA variations: A systematic review and meta‑analysis

**DOI:** 10.3892/mi.2024.178

**Published:** 2024-07-15

**Authors:** George Fotakopoulos, Alexandros G. Brotis, Ourania Fotakopoulou, Charalampos Gatos, Konstantinos Mantzarlis, Vasiliki Epameinondas Georgakopoulou, Pagona Sklapani, Nikolaos Trakas, Kostas N. Fountas

**Affiliations:** 1 Department of Neurosurgery, General University Hospital of Larissa, 41221 Larissa, Greece; 2 Department of Pediatrics, General Hospital of Zakynthos ‘Agios Dionysios’, 29100 Zakynthos, Greece; 3 Department of Critical Care Medicine, University Hospital of Larissa, 41221 Larissa, Greece; 4 Department of Pathophysiology, National and Kapodistrian University of Athens, 11527 Athens, Greece; 5 Department of Biochemistry, Sismanogleio Hospital, 15126 Athens, Greece

**Keywords:** anterior cerebral artery, anatomy A1, anatomy A2, anterior cerebral artery variations, anterior cerebral artery anomalies

## Abstract

The anterior cerebral artery (ACA) and its divisions enclose symptomatically critical and supplementary differentiations. Anatomical variations of the distal ACA that are irregularly detected can be separated into three major groups, namely, azygos, bihemispheric and median ACA variations. The present study performed a systematic review and meta-analysis. The PICOS criteria and electronic databases, namely the Cochrane Library, PubMed (until December, 2023), Embase (until December, 2023) and MEDLINE (until December, 2023) were used to identify 48 articles to fulfill the eligible criteria. As a limited number of studies exist on the prevalence of ACA anatomical variations, the present meta-analysis aimed to determine the precise incidence of these variants. In addition, with the comparative description between cadaveric (autopsy) and imaging cases, more accurate results were extract from the prevalence presentation of the distal ACA variants. On the whole, no statistically significant differences were found between autopsy and imaging studies.

## Introduction

The anterior cerebral artery (ACA) and its divisions enclose symptomatically critical differentiations. Anatomical variations of the distal ACA that are irregularly detected can be separated into three main groups, namely azygos, bihemispheric and median ACA variations (1). The azygos ACA appears after the fusion of the two A2 sections, which pass through the medial wall of the brain and separate under the genus (2-7). In addition, when one of the two A2 divisions is hypoplastic, the contralateral artery separates to irrigate the hemispheres at the same time. This structure is known as a bihemispheric ACA (6,8,9). When an additional third distal ACA branch appears, running to the distal medial surface of one or both hemispheres, this anatomical variant is named median ACA (8,10-12). The acquaintance with the ACA structure is essential for neurosurgeons and radiologists in the identification and managing pathological injuries, although avoiding lesions such as aneurysm development and low irrigation, leading to cerebral ischemia (13).

As the number of available studies on the prevalence of the ACA anatomical variations are limited, the present systematic review and meta-analysis aimed to determine the precise incidence of these variants. In addition, with the comparative description between cadaveric (autopsy) and imaging cases, more accurate results can be extracted from the prevalence presentation of the distal ACA variants.

## Data and methods

### Literature search strategy

The present meta-analysis examined the relative studies involving intracranial ACA variations imaging vs. autopsy evaluation throughout electronic records, counting the Cochrane Library, PubMed (until December, 2023), Embase (until December, 2023), and MEDLINE (until December, 2023). For the study protocol establishment and plan, the Preferred Reporting Items for Systematic Reviews and Meta-Analyses (PRISMA) guidelines were applied. The key words ‘anterior cerebral artery’, ‘Anatomy A1’, ‘Anatomy A2’, ‘anterior cerebral artery variations’ and ‘anterior cerebral artery anomalies’ were used.

### Selection of studies

**Figure 1:**
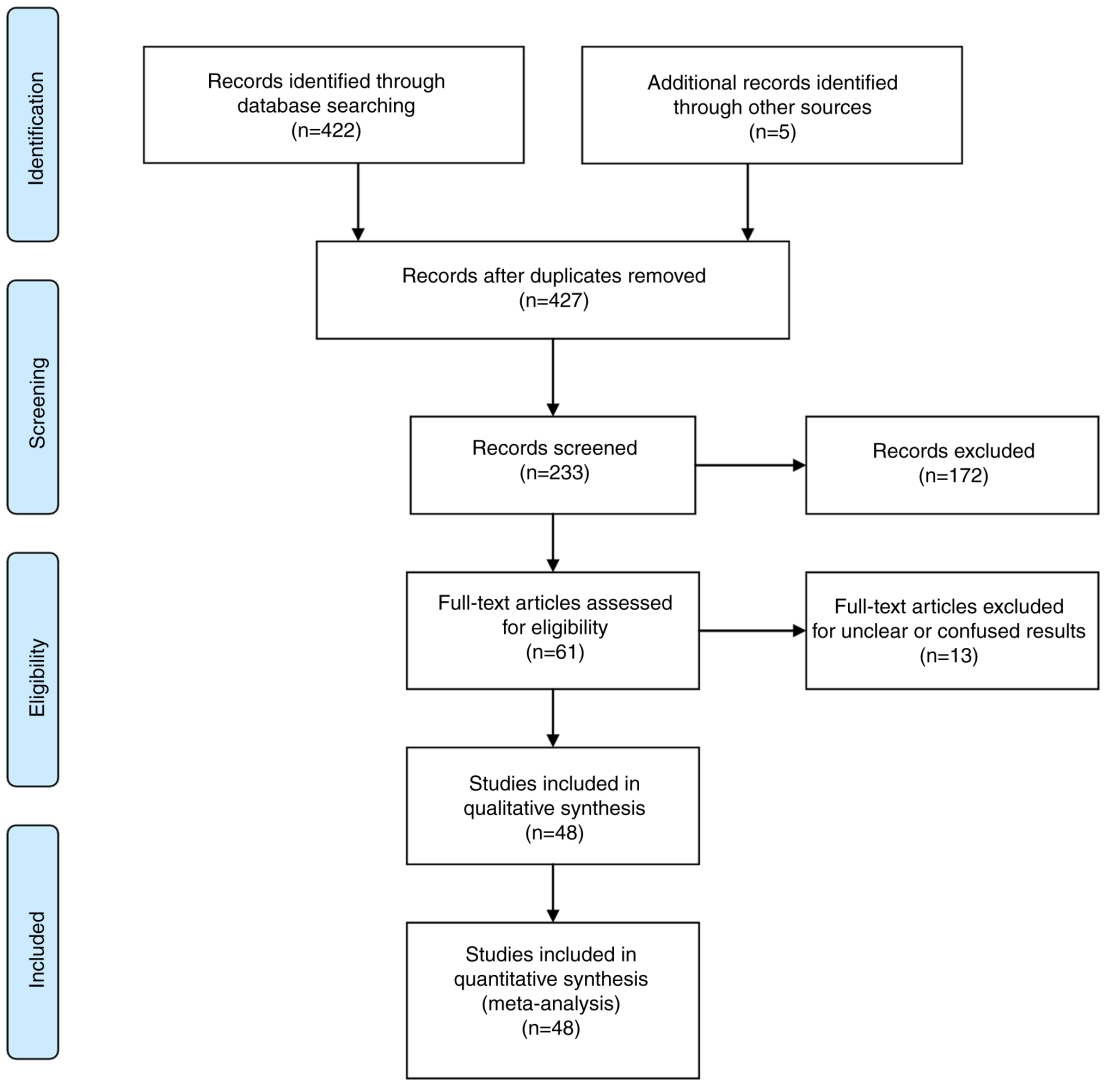
Flowchart of the study selection process.

For the evaluation of the risk of bias, the Cochrane Collaboration tool was applied by two authors (GF and AGB) for each article. The evaluation included random sequence generation and allocation concealment. The assessed results were classified according to the percentage of the risk into low, high or unclear. In the case of a discrepancy, a different investigator with authority provided the concluding solution. The flow chart of the data extraction procedure is presented in Fig. 1.

### Screening

The following exclusion criteria were used: Duplicate articles and those without clear results were excluded from the final article pool. Bibliographic fields, such as title, abstract and investigators were noticeable through the screening. The final article pool excluded duplicate articles and those with no clear results. Records were identified through database searching (n=422 articles) and an additional search through additional bases also identified articles (n=5). Documentations after duplicates were eliminated (n=427). The records were screened (n=233), and records were ruled out (n=172). Full-text articles were evaluated for inclusion criteria (n=61) and eliminated for unclear or confusing results (n=13). The remaining articles were included in the qualitative procedure (n=48). The inclusion criteria were the following: i) Included relative studies involving intracranial ACA variations imaging vs. autopsy evaluation; ii) were primary research articles; and iii) studies published in the English language.

### Extraction process

The following entities were extracted from the selected studies: Estimations of associations between different ACA variations, sample sizes and sample characteristics, the prevalence of each ACA variation, and comparisons between imaging and autopsy data. A total of 48 articles were independently found to fulfill the criteria. There is no test to evaluate the export agreement. The extraction procedures are usual compromises and depend on a large sample of patients (>24.949 patients in the 48 included studies).

For the primary research question, the present study used PICOS criteria (population, intervention, comparison, outcomes and study), to determine eligibility into the article pool. The complete information of these studies is presented in Table I.

Secondary research question(s) were associated with the study design and method (imaging or autopsy).

### Expectations and hypotheses

It was hypothesized that there is a difference between autopsy and imaging studies concerning the prevalence of ACA variations. The variables used were azygos ACA, bihemispheric ACA and median ACA. All prospective and retrospective studies that evaluated these modalities were included. By contrast, reviews, editorials, pediatric cases, case reports, uncertain methods, or one of the two modalities separately from that article pool were excluded. Moreover, in order to reduce the risk of bias in the contained studies, the Newcastle-Ottawa Scale (NOS) was applied as a quality evaluation measurement (Table II) (14).

### Statistical analysis

A random- and fixed-effects form meta-analysis was used to evaluate the proportion estimate for every outcome independently, as the I^2^ statistic was used to calculate the heterogeneity. A value of I^2^ in an amount <50% was considered as low heterogeneity, and an amount >50% was considered as high heterogeneity. The consequences were illustrated on forest plots. The Egger's regression test was used for the calculation of the risk of publication bias. The statistical package R We applied for all statistical analyses (R: Language and Environment, 2010). A value of P<0.05 was considered to indicate a statistically significant difference.

## Results

In total, 48 articles (5-7,10,12,13,15,16-56) fulfilled the eligibility criteria. The entire number of participants was 24,949 [20,399 (81.7%) in imaging and 4,550 (18.3%) in autopsy observed groups]. The study sample was based on 48 articles (5-7,10,12,13,15,16-56) (Table I) and all of these articles were retrospective.

### Azygos ACA variations

**Figure 2:**
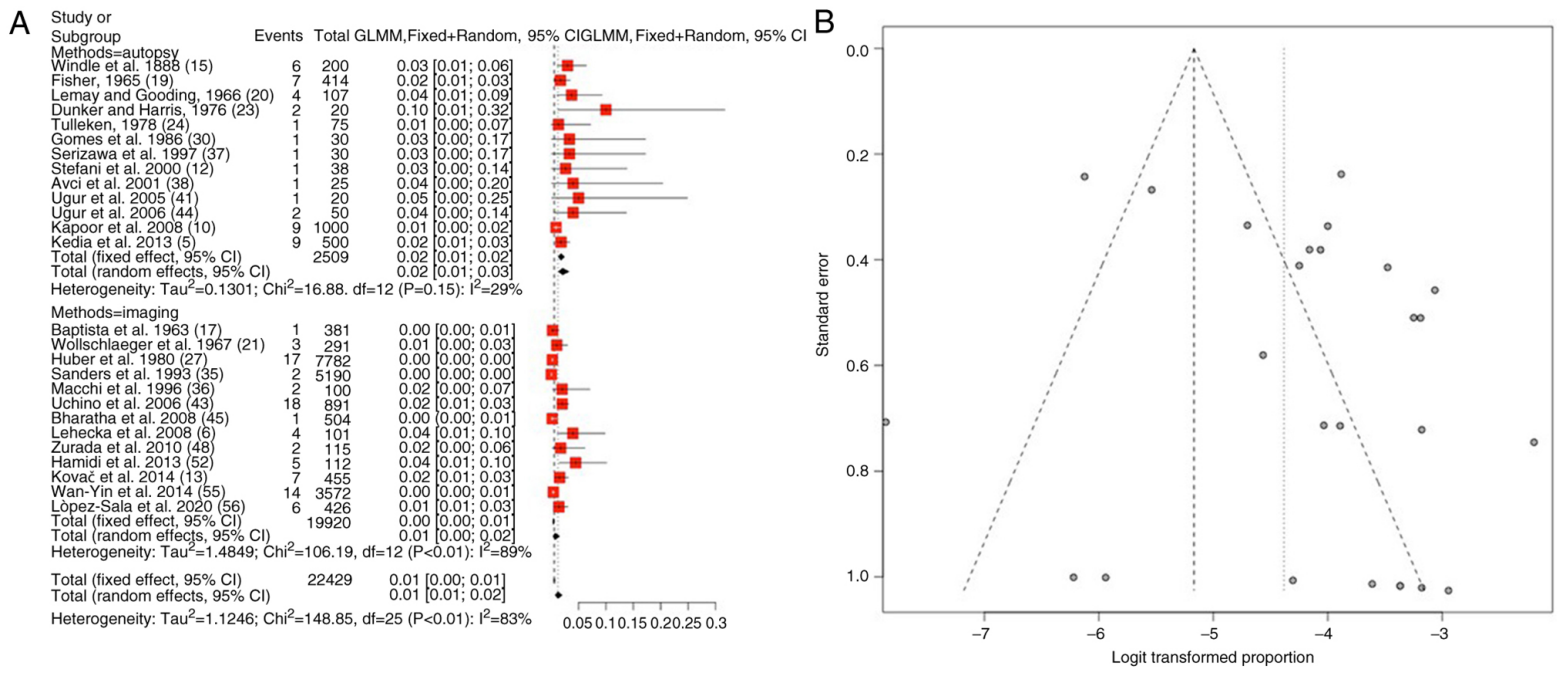
(A) Forest plot for azygos ACA. The results demonstrated that the prevalence of azygos ACA was 1.5% (mean) (95% CI, 0.01-0.02, P<0.01). (B) Funnel plot, testing the sensitivity with funnel plot for azygos ACA; significant publication bias was found (P<0.01) and the heterogeneity was extensive (I^2^=83%). ACA, anterior cerebral artery; I^2^, the percentage of total variation across studies that is due to heterogeneity rather than chance; CI, confidence interval.

Information regarding azygos ACA variations was available in 26 articles (5,6,10,12,13,15,17,19-21,23,24,27,30,35-38,41,43-45,48,52,55,56). The total number of patients was 22,429 [19,920 (88.8%) in imaging and 2,509 (11.2%) in autopsy observed groups]. The prevalence of azygos ACA was 1.5% (mean) (95% CI, 0.01-0.02, P<0.01) (Table III and Fig. 2A). The heterogeneity was extensive (I^2^=83%). When examining the funnel plot, it was established that there was a significant publication bias (P<0.01; Fig. 2B). No significant differences were found between the prevalence established in autopsy (2%) and imaging (1%) studies (Table III).

### Bihemishperic ACA variations

**Figure 3:**
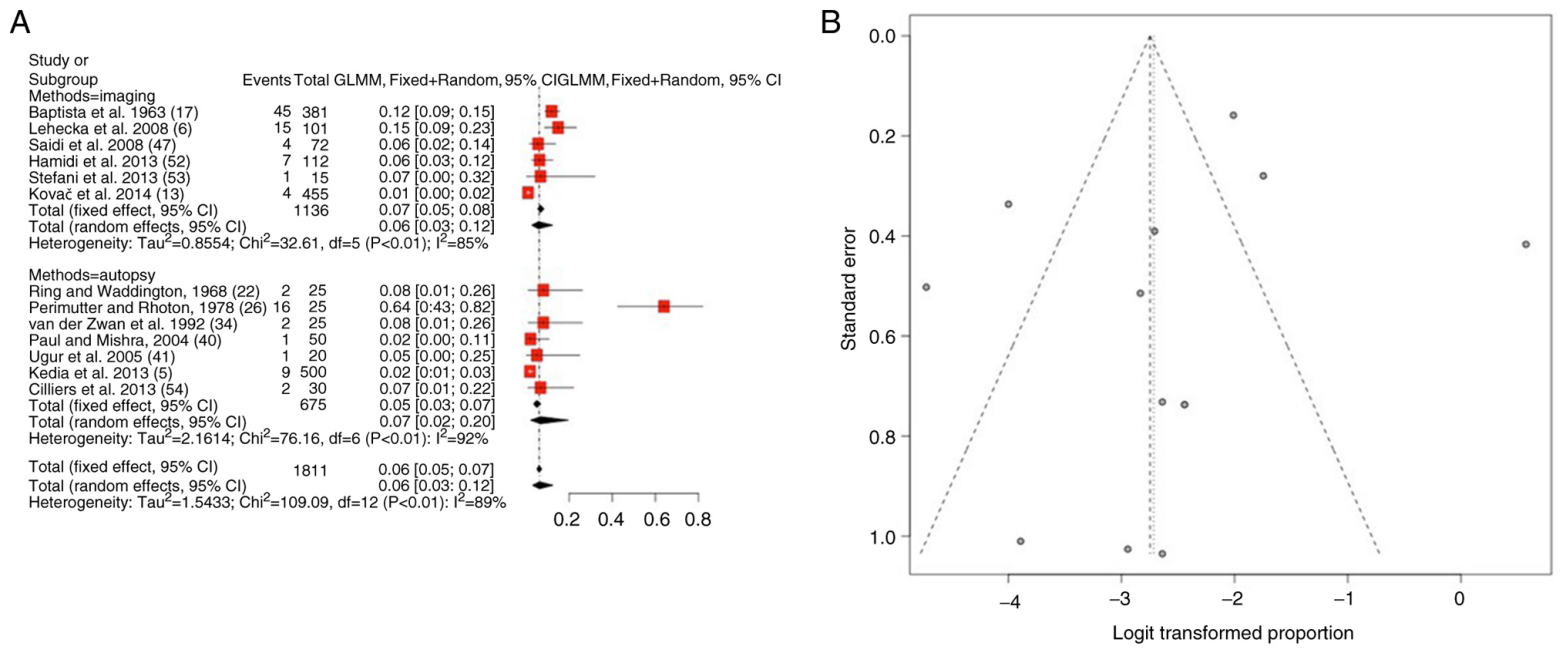
(A) Forest plot for bihemispheric ACA. The results demonstrated that the prevalence of bihemishperic ACA was 7.5% (mean) (95% CI, 0.03-0.12). (B) Funnel plot for bihemispheric ACA; significant publication bias was found (P<0.01) and the heterogeneity was significant (I^2^=89%). ACA, anterior cerebral artery; I^2^, the percentage of total variation across studies that is due to heterogeneity rather than chance; CI, confidence interval.

As regards bihemispheric ACA variations, information was available in 13 articles (5,6,13,17,22,26,34,40,41,47,52-54). The total number of patients was 1,811 [1,136 (62.7%) in imaging and 675 (37.3%) in autopsy-observed groups]. The prevalence of bihemishperic ACA was 7.5% (mean) (95% CI, 0.03-0.12) (Table III and Fig. 3A). The heterogeneity was significant (I^2^=89%). When examining the funnel plot, it was established that there was a high publication bias (P<0.01) (Fig. 3B). No statistically significant differences were found between the prevalence of autopsy (11%) and imaging (7.5%) studies (Table III).

### Median ACA variations

**Figure 4:**
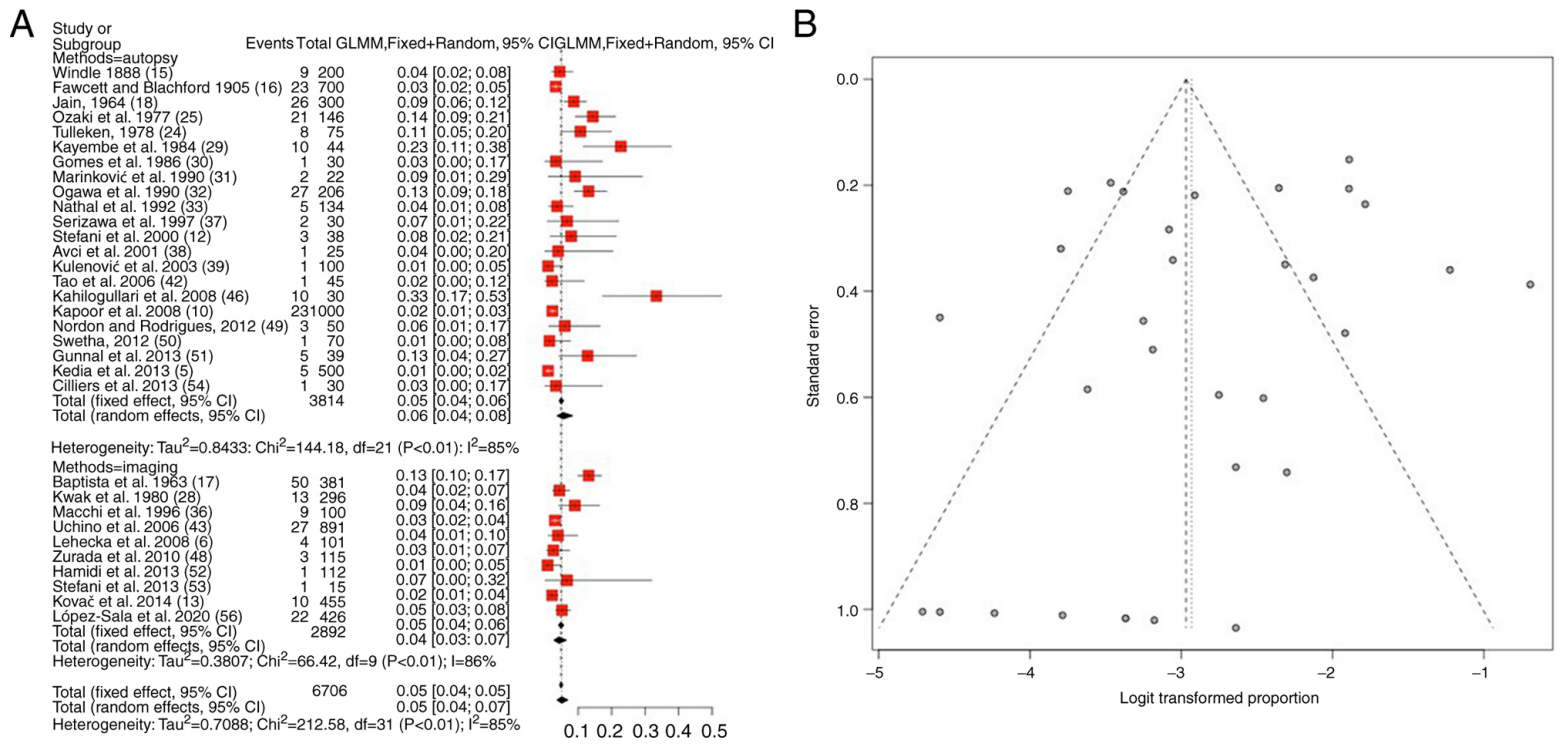
(A) Forest plot for median ACA. The results demonstrated that the prevalence of median ACA was 5.5% (mean) (95% CI, 0.04-0.07, P<0.01). (B) Funnel plot testing the sensitivity with funnel plot for median ACA; significant publication bias was found (P<0.01) and the heterogeneity was considerable (I^2^=85%). No considerable differences were found between the prevalence determined in autopsy (6%) and imaging (5%) studies. ACA, anterior cerebral artery; I^2^, the percentage of total variation across studies that is due to heterogeneity rather than chance; CI, confidence interval.

As regards, median ACA variations, information was available in 32 articles (5,6,10,12,13,15-18,24,25,28-33,36-39,42,43,46,48-54,56). The total number of patients was 6,706 [2,892 (43.1%) in imaging and 3,814 (56.9%) in autopsy observed groups]. The prevalence of the median ACA variant was 5.5% (mean) (95% CI, 0.04-0.07, P<0.01) (Table III and Fig. 4A). The heterogeneity was considerable (I^2^=85%). When examining the funnel plot, it was established that there was a high publication bias (P<0.01) (Fig. 4B). No considerable differences were found between the prevalence evaluated in imaging (5%) and autopsy (6%) articles (Table III).

## Discussion

**Figure 5:**
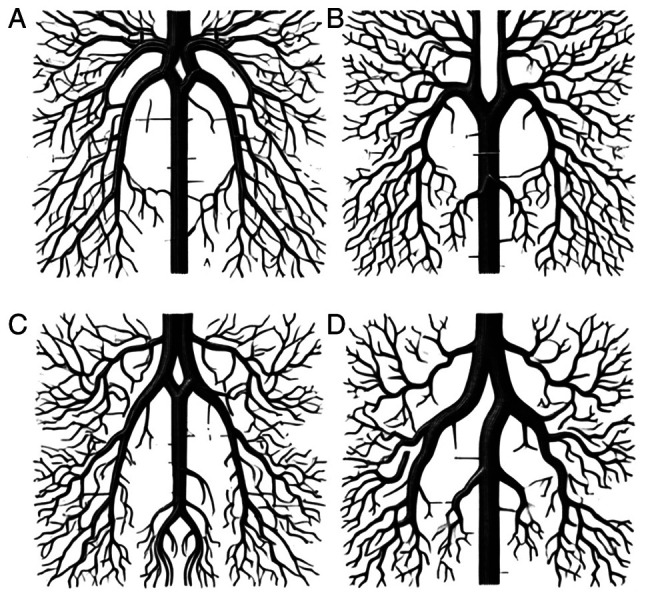
The anatomical variations of the distal ACA. (A) Normal pattern, (B) azygos, (C) median, and (D) bihemispheric ACA variations. ACA, anterior cerebral artery. This image was generated using the ChatGPT artificial intelligence tool.

Anatomical variations of the distal ACA that are irregularly detected can be separated into three main groups, namely azygos, bihemispheric and median ACA variations (1) (Fig. 5). Concerning the topography and morphology, the azygous ACA variation reveals a particular midline vessel created from the connection of bilateral A1 segments next to the typical locality of the anterior communicating artery (A-comm) (20). Thus, mainly the A-comm is mislaid or hypoplastic, and the formed midline vessel passes through the inter-hemispheric fissure, supplying the medial hemispheres with blood (20). The clinical interest of the azygous ACA is that its appearance consists of pathologies leading to infarcts or aneurysms (57,58). According to the literature, the occurrence of an azygous ACA is 0.3% (2,59). The present meta-analysis revealed that the prevalence of azygos ACA was 1.5% [autopsy (2%) and imaging (1%)].

Another moderately comparable anatomic modification is the bihemispheric ACA, where one of the two contralateral A1 segments is hypoplastic (59). Thus, the bihemispheric ACA feeds the two pericallosal regions equally with blood and its one-sided callosomarginal region (59). A with the azygos ACA, the bihemispheric ACA variation is connected with a number of pathologies, such as infarcts and aneurysms, in the regions where it supplies (59,60). In the literature, the prevalence of the bihemispheric ACA variation was found to be 0.20-8.0% (5,27). The present meta-analysis demonstrated that the prevalence of bihemishperic ACA was 7.5%, and no significant differences were found between the prevalence in autopsy (11%) and imaging (7.5%) studies.

Strongly related to the azygos ACA is an additional variant where a median ACA is detected, and the third distal ACA appearance divisions to the distal medial region of one or both hemispheres (8,10,11). This variation may be the result of a hypoplastic ACA and the persistent expansion of the median artery of the corpus callosum (61). The literature demonstrates a wide range in the prevalence of median ACA between 1.0 and 35.0% (5,48). The present study revealed that the median ACA variant was 5.5%, and there were no notable differences between the prevalence evaluated in imaging (5%) and autopsy (6%) articles.

The present meta-analysis had certain limitations that should be mentioned. The main inadequacy was that its retrospective character was associated with potential miscalculations in assembling and understanding the records from the medical history.

In conclusion, the variations of the ACA's provide significant blood supply to anatomically valuable regions, such as the corpus callosum, or frontal lobe and basal ganglia. In addition, the pathologies behind their appearance, such as infarcts or aneurysm development, are critical. Thus, the knowledge of the ACA variations in prevalence may aid clinicians in managing aneurysms or tumors and other surgical procedures involving these regions, providing a strong justification for more extensive prospective clinical investigations.

## Figures and Tables

**Table I:** Determined prevalence of anatomical characteristics based on study type (autopsy or imaging).

A, Autopsy						
Author(s), year of publication	Study design	Total no. of patients, n=24, 949 (100%)	Azygos ACA, n=127/22, 429 (0.6%)	Bihemishperic ACA, n=109/1, 811 (6.0%)	Median ACA, n=319/6, 706 (4.7%)	(Refs.)
Windle *et al*, 1888	Retro	200	6	0	9	(15)
Fawcett and Blachford, 1905	Retro	700	0	0	23	(16)
Jain, 1964	Retro	300	0	0	26	(18)
Fisher, 1965	Retro	414	7	0	0	(19)
LeMay and Gooding, 1966	Retro	107	4	0	0	(20)
Ring and Waddington, 1968	Retro	25	0	2	0	(22)
Dunker and Harris, 1976	Retro	20	2	0	0	(23)
Ozaki *et al*, 1977	Retro	146	0	0	21	(25)
Perlmutter and Rhoton, 1978	Retro	25	0	16	0	(26)
Tulleken, 1978	Retro	75	1	0	8	(24)
Kayembe *et al*, 1984	Retro	44	0	0	10	(29)
Gomes *et al*, 1986	Retro	30	1	0	1	(30)
Marinković *et al*, 1990	Retro	22	0	0	2	(31)
Ogawa *et al*, 1990	Retro	206	0	0	27	(32)
Nathal *et al*, 1992	Retro	134	0	0	5	(33)
van der Zwan *et al*, 1992	Retro	25	0	2	0	(34)
Serizawa *et al*, 1997	Retro	30	1	0	2	(37)
Stefani *et al*, 2000	Retro	38	1	0	3	(12)
Avci *et al*, 2001	Retro	25	1	0	1	(38)
Kulenović *et al*, 2003	Retro	100	0	0	1	(39)
Paul and Mishra, 2004	Retro	50	0	1	0	(40)
Ugur *et al*, 2005	Retro	20	1	1	0	(41)
Tao *et al*, 2006	Retro	45	0	0	1	(42)
Ugur *et al*, 2006	Retro	50	2	0	0	(44)
Kahilogullari *et al*, 2008	Retro	30	0	0	10	(46)
Kapoor *et al*, 2008	Retro	1,000	9	0	23	(10)
Nordon and Rodrigues, 2012	Retro	50	0	0	3	(49)
Swetha, 2012	Retro	70	0	0	1	(50)
Gunnal *et al*, 2013	Retro	39	0	7	1	(51)
Kedia *et al*, 2013	Retro	500	9	1	1	(5)
Cilliers *et al*, 2018	Retro	30	0	0	5	(54)
B, Imaging						
Author(s), year of publication	Study design	Total no. of patients, n=24, 949 (100%)	Azygos ACA, n=127/22, 429 (0.6%)	Bihemishperic ACA, n=109/1, 811 (6.0%)	Median ACA, n=319/6, 706 (4.7%)	(Refs.)
Baptista *et al*, 1963	Retro	381	1	45	50	(17)
Wollschlaeger *et al*, 1967	Retro	291	3	0	0	(21)
Huber *et al*, 1980	Retro	7,782	17	0	0	(27)
Kwak *et al*, 1980	Retro	296	0	0	13	(28)
Sanders *et al*, 1993	Retro	5,190	2	0	0	(35)
Macchi *et al*, 1996	Retro	100	2	0	9	(36)
Uchino *et al*, 2006	Retro	891	18	0	27	(43)
Bharatha *et al*, 2008	Retro	504	1		0	(45)
Lehecka *et al*, 2008	Retro	101	4	15	4	(6)
Saidi *et al*, 2008	Retro	72	0	4	0	(47)
Nowinski *et al*, 2009	Retro	96	0	0	0	(7)
Zurada *et al*, 2010	Retro	115	2	0	3	(48)
Stefani *et al*, 2013	Retro	15	0	2	1	(53)
Hamidi *et al*, 2013	Retro	112	5	9	5	(52)
Kovač *et al*, 2014	Retro	455	7	4	10	(13)
Wan-Yin *et al*, 2014	Retro	3,572	14	0	0	(55)
López-Sala *et al*, 2020	Retro	426	6	0	22	(56)

ACA, anterior cerebral artery; Retro, retrospective.

**Table II:** Newcastle-Ottawa Scale (NOS) quality assessment of final article pool.

A, Autopsy						
		Newcastle-Ottawa Scale				
Author(s), year of publication	Study design	Selection	Comparability	Exposure	Total scores	(Refs.)
Windle *et al*, 1888	Retro	3	3	3	9	(15)
Fawcett and Blachford, 1905	Retro	3	3	3	9	(16)
Jain, 1964	Retro	3	3	3	9	(18)
Fisher, 1965	Retro	3	3	3	9	(19)
Lemay and Gooding, 1966	Retro	3	3	3	9	(20)
Ring and Waddington, 1968	Retro	3	3	3	9	(22)
Dunker and Harris, 1976	Retro	2	2	3	7	(23)
Ozaki *et al*, 1977	Retro	2	2	3	7	(25)
Perlmutter and Rhoton, 1978	Retro	2	3	3	8	(26)
Tulleken, 1978	Retro	3	3	3	9	(24)
Kayembe *et al*, 1984	Retro	3	3	3	9	(29)
Gomes *et al*, 1986	Retro	3	3	3	9	(30)
Marinković *et al*, 1990	Retro	3	3	3	9	(31)
Ogawa *et al*, 1990	Retro	3	3	3	9	(32)
Nathal *et al*, 1992	Retro	3	3	3	9	(33)
van der Zwan *et al*, 1992	Retro	3	3	3	9	(34)
Serizawa *et al*, 1997	Retro	3	3	3	9	(37)
Stefani *et al*, 2000	Retro	3	3	3	9	(12)
Avci *et al*, 2001	Retro	3	3	3	9	(38)
Kulenović *et al*, 2003	Retro	3	3	3	9	(39)
Paul and Mishra, 2004	Retro	3	3	3	9	(40)
Ugur *et al*, 2005	Retro	3	2	3	8	(41)
Tao *et al*, 2006	Retro	3	3	2	8	(42)
Ugur *et al*, 2006	Retro	3	3	3	9	(44)
Kahilogullari *et al*, 2008	Retro	3	3	3	9	(46)
Kapoor *et al*, 2008	Retro	3	3	3	9	(10)
Nordon and Rodrigues, 2012	Retro	3	3	3	9	(49)
Swetha, 2012	Retro	3	3	3	9	(50)
Gunnal *et al*, 2013	Retro	3	3	3	9	(51)
Kedia *et al*, 2013	Retro	3	3	3	9	(5)
Cilliers *et al*, 2018	Retro	3	3	3	9	(54)
B, Imaging						
		Newcastle-Ottawa Scale				
Author(s), year of publication	Study design	Selection	Comparability	Exposure	Total scores	(Refs.)
Baptista *et al*, 1963	Retro	3	2	3	8	(17)
Wollschlaeger *et al*, 1967	Retro	3	3	3	9	(21)
Huber *et al*, 1980	Retro	3	3	3	9	(27)
Kwak *et al*, 1980	Retro	3	3	3	9	(28)
Sanders *et al*, 1993	Retro	3	3	3	9	(35)
Macchi *et al*, 1996	Retro	3	3	3	9	(36)
Uchino *et al*, 2006	Retro	3	3	3	9	(43)
Bharatha *et al*, 2008	Retro	3	3	3	9	(45)
Lehecka *et al*, 2008	Retro	3	3	3	9	(6)
Saidi *et al*, 2008	Retro	3	3	3	9	(47)
Nowinski *et al*, 2009	Retro	3	3	3	9	(7)
Zurada *et al*, 2010	Retro	3	3	3	9	(48)
Stefani *et al*, 2013	Retro	3	3	3	9	(53)
Hamidi *et al*, 2013	Retro	3	3	3	9	(52)
Kovač *et al*, 2014	Retro	3	3	3	9	(13)
Wan-Yin *et al*, 2014	Retro	3	3	3	9	(55)
López-Sala *et al*, 2020	Retro	3	3	3	9	(56)

Retro, retrospective.

**Table III:** Parameters for the results of the meta-analysis.

		Groups		Overall effect		Heterogeneity		Prevalence (%)	
Parameters	Included Trials (n=48)	Imaging	Autopsy	Effect estimate	95% CI	I^2^ (%)	P-value	Imaging	Autopsy
Azygos	26	19920	2509	0.01	(0.01-0.02)	83	<0.01	2	1
Bihemishperic	13	1136	675	0.01	(0.03-0.12)	89	<0.01	7.5	11
Median	32	2892	3814	0.05	(0.04-0.07)	85	<0.01	5	6

I^2^, the percentage of total variation across studies that is due to heterogeneity rather than chance; CI, confidence interval.

## Data Availability

The datasets used and/or analyzed during the current study are available from the corresponding author on reasonable request.
